# Lattice add-ons with low-content Pd incorporated into Pt nanodendrites revealed active methanol oxidation *via* the fast removal of poisonous intermediates

**DOI:** 10.1039/d5na00672d

**Published:** 2025-10-13

**Authors:** Ammar Bin Yousaf, Asad Ali, Peter Kasak

**Affiliations:** a Center for Advanced Materials, Qatar University Doha 2713 Qatar ammar@mail.ustc.edu.cn peter.kasak@qu.edu.qa; b School of Materials Science and Engineering, Hefei University of Technology Hefei 230009 P. R. China

## Abstract

Methanol-driven fuel cells experience challenges in their performance due to the complex mechanism for the complete conversion of reactants into products. The electrocatalysts undergo partial or strong adsorption of poisonous intermediates during methanol oxidation, which hinders their activity. In this regard, we have designed a facile strategy to develop a catalyst with enhanced and stable methanol electro-oxidation at an economical cost value. The incorporation of lattice add-ons with low-content Pd into Pt nanodendrites (NDs) *via* the co-reduction single-step method was successfully employed. These Pd-coupled Pt NDs were uniformly dispersed and loaded on reduced graphene oxide (RGO). The PtPd NDs/RGO catalyst exhibited 4 times greater methanol oxidation reaction performance (2 mA cm^−2^) compared to the commercial Pt/C catalyst. Time-resolved *in situ* Fourier transform infrared (FTIR) spectroscopy and online differential electrochemical mass spectrometry (DEMS) revealed that the methanol oxidation reaction (MOR) followed the active intermediate pathway for its complete conversion into the final product and proceeded through a six-electron transfer reaction.

## Abstract 2

Lattice add-ons with low-content Pd incorporated into Pt nanodendrites enabled active methanol oxidation *via* the fast removal of poisonous intermediates.

## Graphical abstract

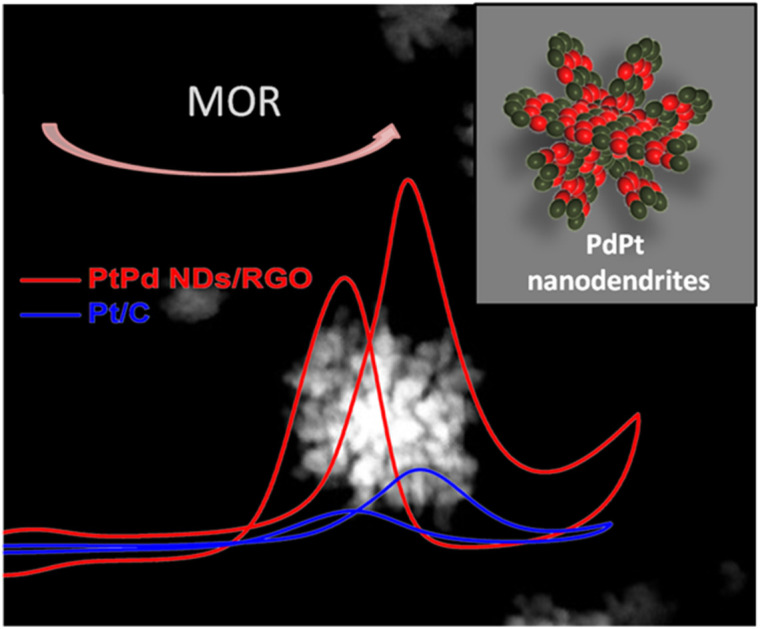

## Introduction

Methanol oxidation reactions (MORs) are carried out mainly by Pt and Pt-based catalysts in commercial fuel cells.^1,2^ The mechanism of methanol chemisorption remains critical due to the formation of several intermediate species. Among these intermediates, the CO intermediate causes catalyst surface poisoning. This poisoning species adsorbs on the metallic surface of the catalyst as CO_ads_ and prevents the further adsorption of methanol molecules on the surface for chemisorption.^3,4^ This poisoning phenomenon is one of the main reasons that the enhancement of direct methanol fuel cell technology has been hindered. Several strategies have been adopted to overcome the CO-poisoning issues and improve the performance of Pt-based catalysts, such as alloying Pt with its group elements (*i.e.*, Pd, Ag, Au, and Ru).^5–8^ Alloying Pt with additional elements might not only exhibit benefits such as anti-poisoning effects but also show specific adverse effects. The alloying of noble metals, *i.e.*, Ag, Au and Ru with Pt, experienced stability issues due to the dissolution of active sites.^9,10^ The stability is improved in the presence of Pd alloyed with Pt due to favourable bonding between Pt and Pd in the alloy, which significantly reduces the factor of active site dissolution during methanol oxidation reactions.^10,11^ Thus, with the adoption of several Pt-based combinations, Pd has emerged as a promising choice for developing high-performance catalysts. In this combination, Pd assists in the smooth oxidation of methanol by following the bifunctional mechanism.^12–14^ Pd, as an alloying element, mainly facilitates adsorbed oxygen-containing species, which enhance the methanol oxidation intermediates, predominantly CO to CO_2_. Pd also modifies the electronic structure of Pt at the orbital level after alloying with Pd, *i.e.*, there is a shift in the d-bands.^15,16^ This modification in the Pt d-bands weakens the strong adsorption of the CO_ads_ intermediate species on the Pt-active sites and leads to weak adsorptions. Therefore, the weakly adsorbed CO_ads_ is subsequently removed faster from the surface by reacting with nearby OH_ads_ species converting it into non-poisoning species and to the final product of MOR.^12–16^

In addition to the involvement of metallic catalysts in the MOR, the role of support materials remains accountable. As it is proposed, the MOR is a six-electron transfer reaction.^17^ The mechanism of the MOR may strongly influence the electron transfer phenomenon facilitated by the substrate surface in the catalyst.^18^ Among those, carbonaceous support materials have expectedly provided a number of benefits while working as substrate surfaces for catalysts. The high dispersion of the metal catalysts can favourably be achieved on these carbonaceous supports. The strong coupling of the metal particles with enhanced metal-to-support affinities can be targeted. Due to the strong interactions of the metal catalyst particles with the carbonaceous support, the enhanced electron transfer phenomenon generated within the catalyst material facilitates the six-electron transfer reactions of the MOR.^19–22^ The combination of alloys and suitable support materials introduces a synergy effect, where the incorporation of Pt with Pd in the alloy by bifunctional mechanism facilitates the continuous adsorption of methanol molecules on the catalyst surface with the quick release of CO_ads_ from the active sites while the support materials enhance the metal to support the electron transfer reaction.^8^

Efforts were previously made to introduce Pt–Pd nanodendrite catalysts for the methanol oxidation reaction.^12,14^ In all previous reports, simple fabrications of Pt–Pd nanodendrites were done with an emphasis on the design of bimetallic catalyst materials favourable for the methanol oxidation reaction. Thus, in this work, we have specifically focused on further structural engineering that involves the incorporation of low-content Pd into the lattice of Pt nanodendrites. For the first time, the concept of lattice add-ons has been introduced in monometallic shaped Pt-based catalysts in order to develop low-cost bimetallic Pt-group alloys. The lattice add-ons concept in this work strategically involves the modification of the lattice structure, electronic properties and surface chemistry of the core Pt nanodendrites by depositing Pd atoms on its surfaces. This technique is primarily adopted to enhance the catalytic activity, durability and cost-effectiveness of the electrocatalysts for electrochemical reactions. More specifically, this work has targeted the synthesis of PtPd nanodendrites (NDs) with the mixed-phase metallic structure on the surface of reduced graphene oxide (RGO). Incorporation is achieved by first aligning the lattice of the core Pt, and the Pd is then set on or integrated with the Pt surface following a crystallographically controlled method. The Pt and Pd in their mixed-phase structure as high-indexed NDs are expected to play a significant role in overcoming the slow kinetics and poisoning issues of the MOR. At the same time, the reduced graphene oxide (RGO) contributes to higher dispersion and strong encoring of the PtPd NDs on their surface. The successful implementation of the effective strategies in designing the catalysts was verified by means of *in situ* spectroelectrochemical studies such as *in situ* Fourier transform infrared (FTIR) and online differential electrochemical mass spectrometry (DEMS) to understand the mechanism of the reaction and formation of the targeted final product. The obtained results with straightforward enhanced performance of the catalysts may be ascribed to the structural and electronic effects of the catalyst material.

## Experimental

This part is provided in the SI file.

## Results and discussions

**Fig. 1:**
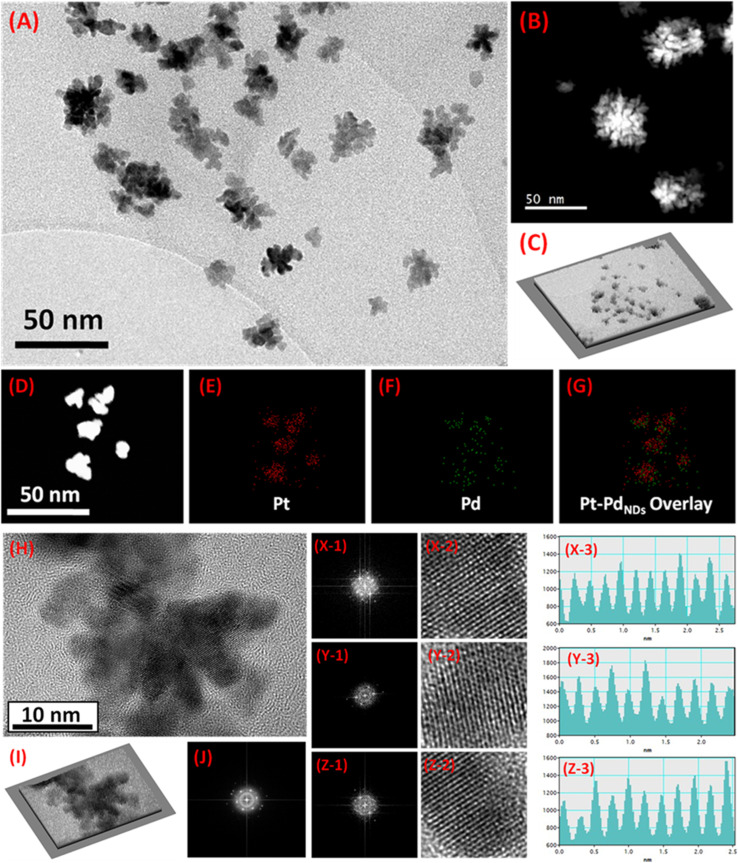
(A) TEM image of PtPd NDs/RGO. (B) Dark-field TEM image of PtPd NDs/RGO. (C) Three-dimensional (3D) surface plot hill–stack plots for PtPd NDs loaded on RGO. (D) Dark-field image of the selected area for elemental mapping analysis for PtPd NDs/RGO with the specific area considered for the presence of: (E) Pt, (F) Pd and (G) the combined overlayered image. (H) HRTEM image of PtPd NDs/RGO; (I) three-dimensional (3D) surface plot hill–stack plots for PtPd NDs/RGO extracted from the corresponding HRTEM micrograph; (J) selected area electron diffraction (SAED) pattern for PtPd NDs/RGO. The corresponding fast Fourier transform (FFT) patterns (X-1, Y-1, and Z-1), inverse FFT (X-2, Y-2, and Z-2) and corresponding line histogram profiles (X-3, Y-3, and Z-3) for the lattice plane of (200) (X-1, 2, and 3), (220) (Y-1, 2, and 3) and (111) (Z-1, 2, and 3) in PtPd NDs/RGO.

The PtPd ND alloys on RGO were successfully synthesized by co-reduction single-step synthesis and confirmed by characterizations. The catalyst geometry was designed to incorporate a low content of Pd into the lattice of Pt ND loaded on RGO. In this way, the cost and performance of the obtained material was mainly focused on developing a methanol oxidation reaction catalyst. In materials synthesis, it is important to understand the growth mechanism of the catalyst structures and alloy geometry.^23,24^ For the current work, the general growth mechanism of the PtPd nanodendrites can be described as the nucleation and seed formation of Pt and Pd on graphene oxide, followed by the dendritic growth and reduction of PtPd alloy crystals in the presence of their low-content precursor. In particular, the graphene oxide provided the nucleation and seed growth support for PtPd during the synthesis. Alternatively, following the more common trend, Pt seeds are first grown according to their standard reduction potential (*E*^0^ = +1.20 V) compared to the standard reduction potential of Pd (*E*^0^ = +0.95 V) and serve as nucleation sites for the Pd to be grown on the lattice of Pt-sites. The controlled low-content incorporation of Pd into Pt was achieved according to this mechanistic pathway with the availability of lower precursors for both metals (*i.e.*, Pt > Pd). In the final step, the stabilized growth of PtPd seeds assisted by the CTAB stabilizer on graphene oxide was reduced by l-ascorbic acid as the reducing agent. As the concept of lattice add-ons onto Pt dendrites has been put forwarded, the atomic ratios of Pt and Pd were also tuned by varying the atomic (%) of Pd only to maintain the cumulative 5 wt% Pt–Pd optimized bimetallic alloy. Among different synthesized and tested PtPd alloys ND, the alloys with 1 wt% of Pd and 4 wt% of Pt were screened out as the optimized catalyst and used for detailed physicochemical and electrochemical characterizations. Moreover, the detailed analysis for nominal and actual weight ratios of PtPd nanodendrite alloys has been provided in Table S1 (in the SI file). Transmission electron microscopy (TEM) and high-resolution transmission electron microscopy (HRTEM) was performed to study the morphologies and structural confirmation, respectively. The combined images from the bright-field TEM image (Fig. 1A) and the dark-field TEM image (Fig. 1B) exhibit the growth of dendrites on the RGO surface. The surface layer of the RGO sheets is not specifically visible in the TEM image, but can be clearly seen in the low-magnification TEM image provided in Fig. S1A (in the SI file). The PtPd ND are strongly incorporated into the RGO sheets to develop strong anchoring effects with the support of the catalyst. These features are specifically found by analyzing the obtained TEM images to process the three-dimensional (3D) surface plot hill–stack plots, shown in Fig. 1C. The white grass-like feature corresponds to the RGO and the holes on its surface represent the incorporation of PtPd ND.^25^ The hill–stack plot in Fig. 1C can also ascribed to the surface roughness of the RGO with strongly anchored PtPd ND on its surface.^26^ The good conductive behaviour of the carbonaceous support material depends on the surface roughness and the number of defects on its surface. The obtained 3D surface plot (hill–stack view) can be correlated with the good conductive behaviour of the catalyst support with the uneven hills of the graphene layer having curly edges, sharp spikes and enhanced defect densities with the presence of PtPd ND on it.

Furthermore, element mapping analysis was done to analyze the atomic dispersion of Pt and Pd in the PtPd ND alloy. Fig. 1D from the dark-field TEM image was taken as the selected area for element mapping analysis. The mapping analysis for Pt Fig. 1E, Pd Fig. 1E and overlayered image Fig. 1G showed that Pt and Pd exhibit the homogeneously intermixed atomic dispersion characteristics in the alloy formation. The HAADF-STEM element mapping characterizations were also extended to analyze the catalysts with different Pd wt% for better comparison among the best performance catalysts, with the results shown in Fig. S1B and C (in the SI file). These results show the better intermixed Pt and Pd atomic dispersion in the catalyst with 1 wt% Pd (Fig. 1E–G) compared to those of 2 wt% Pd (Fig. S1B) and 3 wt% Pd (Fig. S1B) catalysts. This trend suggests the feasible lattice add-ons phenomenon among Pt and Pd in the optimized composition PtPd NDs/RGO catalyst, which later favourably influenced the performance of the catalyst in the electrocatalytic reaction. In addition, the intimate contact of the PtPd ND with the RGO surface and their lattice information were characterized by HRTEM analysis. The HRTEM analysis confirms the synthesis of the homogenous-phase PtPd ND with the metallic characteristics exhibiting lattice fringes on the surface of the dendrites,^13,27^ as shown in Fig. 1H. The 3D surface plot (hill–stack view) for the obtained HRTEM image was also processed and is provided in Fig. 1I, showing the significantly incorporated ND (in dark color) on the RGO sheet (with exposed white grass-like spikes from RGO) surface. This characteristic confirmation may also be ascribed to the strongly anchored intimately contacted PtPd ND on RGO sheets. The associated selected area electron diffraction (SAED) pattern image obtained from the HRTEM micrograph exhibited the polycrystalline character of the PtPd dendritic alloys, as depicted in Fig. 1J. The HRTEM analysis was further elaborated with the derivation of fast Fourier transform (FFT) patterns (Fig. X-1, Y-1 and Z-1) and the inverse of these FFT images (Fig. X-2, Y-2 and Z-2). The corresponding line histogram profiles for the corresponding selected lattice fringes were also derived and provided in Fig. X-3, Y-3 and Z-3 to calculate the interplanar (d)-spacing.^28^ The FFT patterns taken at three different proportions from the HRTEM micrograph images gave lattice spacing values of 0.201 nm (Fig. X-2), 0.243 nm (Fig. Y-2) and 0.230 nm (Fig. Z-2) that correspond to the (200) (Fig. X-1), (220) (Fig. Y-1) and (111) (Fig. Z-1) lattice planes, respectively.^13,27–29^

**Fig. 2:**
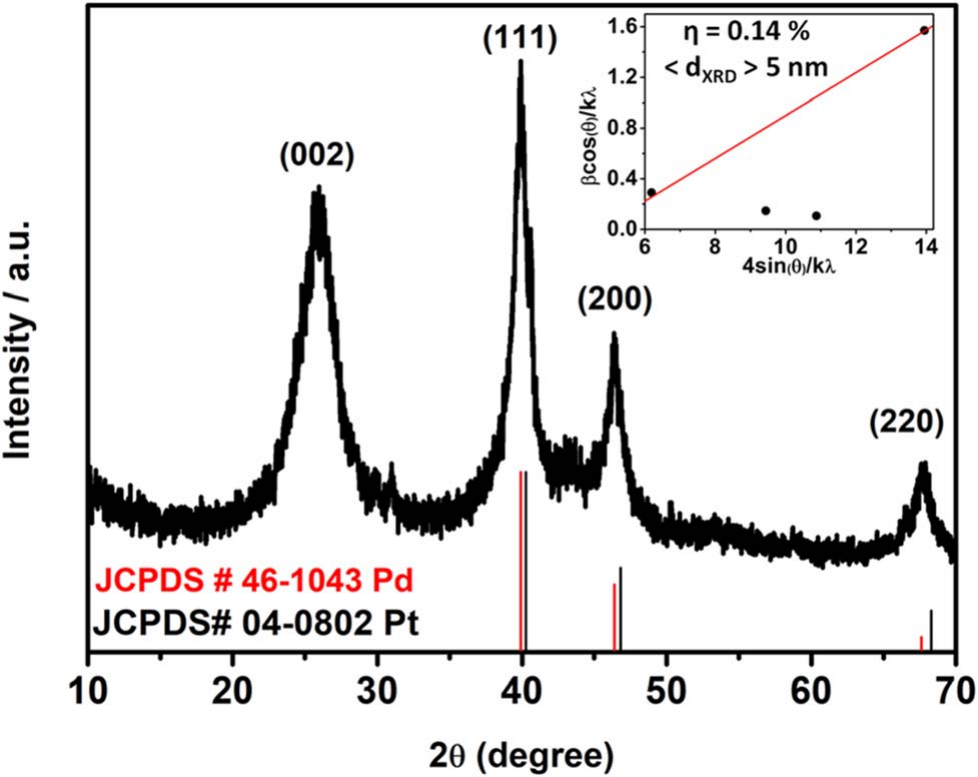
XRD pattern of the PtPd NDs/RGO catalysts, alongside JCPDS standard cards for Pt (black pattern) and Pd (red pattern), inset; the W–H plot derived from the respective XRD spectrum with the calculated lattice strain (*η*) and crystallite size (d) values.

The crystalline and structural phase of PtPd NDs/RGO was characterized by X-ray diffraction (XRD) analysis, presented in Fig. 2. The obtained XRD spectrum for the PtPd ND alloy was compared with the standard JCPDS peaks of monometallic Pt (JCPDS, card no 04-0802) and Pd (JCPDS, card no. 46-1043) to confirm the alloy formation of both elements.^30,31^ The XRD peaks of the PtPd alloy NDs mainly contain the signature features of mixed Pt and Pd, with a slight shift of the corresponding peaks towards higher angles than that of their monometallic form. This phenomenon suggests the successful formation of the Pt and Pd alloy in the ND structure. The characteristic peaks in XRD located at 40.0°, 46.6° and 68.1° correspond to the (111), (200) and (220) planes of the Pt and Pd alloy, respectively.^30,31^ The peaks for the individual Pt and Pd in alloy formation could not be distinguished and appeared to overlap because of the high lattice match of (∼99%) between both elements.^32^ Notably, the lattice planes observed in the XRD results are closely matched, supporting the FFT, inverse FFT and line histogram profiles derived from HRTEM analysis (Fig. X-1–Z-1). Moreover, the RGO in the catalyst was confirmed with a major broader peak at 25.9°, corresponding to the (002) planes from RGO. The XRD spectrum was further analyzed to draw the Williamson Hall plot (W–H plot) for the calculation of the proposed crystalline size and lattice strain. The proposed lattice strain helps to determine the quantification of the induced micro strain in the structure due to the defect points or lattice dislocations after lattice perturbations.^33,34^ In the case of the present work, the lattice perturbations occurred due to the lattice add-ons on the Pt nanodendrites with the incorporation of Pd. The calculation for the drawn W–H plot from the XRD spectrum, shown in the inset of Fig. 2, gave proposed values of (<5 nm>) for the crystalline size and a lattice strain of 0.14%. The observed value of 0.14% lattice strain indicates a slight distortion in the crystalline structure of the nanodendrites, which was induced due to the incorporated Pd into the Pt lattice sites. This calculated proof also strengthens the claim of lattice add-ons on Pt, as there were no additional peaks for the Pd proportion observed in the XRD spectrum. Meanwhile, the micro lattice strain confirmed the addition of Pd into the Pt nanodendrites in the catalyst material.

**Fig. 3:**
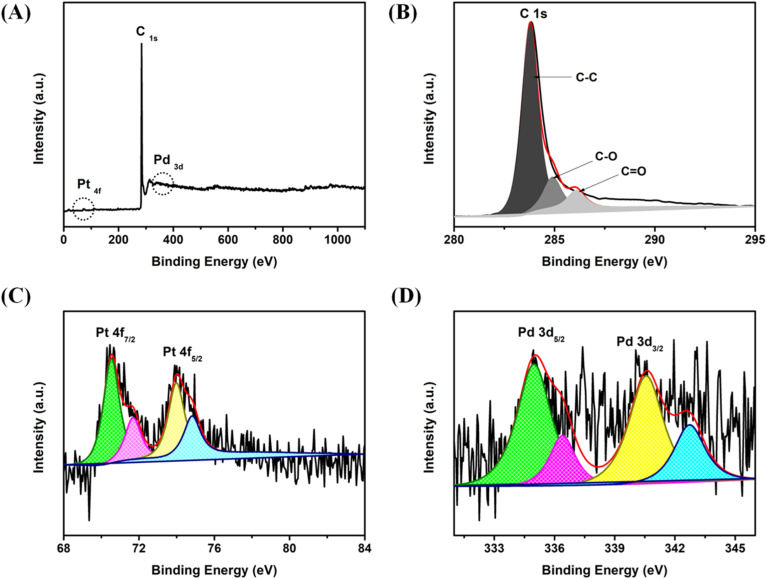
(A) XPS full survey scan of the PtPd NDs/RGO catalyst; (B) C 1s, (C) Pt 4f and (D) Pd 3d high-resolution XPS scans of the PtPd NDs/RGO catalyst.

X-ray photoelectron spectroscopy (XPS) analysis of the PtPd NDs/RGO material was performed to investigate the surface chemistry information and the valence states of the elements. The survey XPS spectrum revealed the presence of C (from RGO) and Pt, Pd (from NDs alloys) at their corresponding binding energy positions (Fig. 3A). The presence of reducing surface groups on the RGO sheets was confirmed by the negligible presence of oxygen in the survey XPS and with the carbon C 1s XPS scan. The C 1s peak contains predominantly C–C bond with theπ—π* bond alongside a significant carbonyl bond (C=O) and (C–O), as shown in Fig. 3B.^35,36^

The peak intensity can be associated with C–C bonds at 283.80 eV, indicating the sp^2^-hybridized graphitic structure. This phenomenon indicates the substantial reduction of oxygenated functional groups with the anchoring of PtPd ND on the RGO sheets. For the XPS scan associated with Pt, the Pt 4f spectrum exhibited a relatively asymmetric nature containing the dominant metallic Pt(0) state with 4f_5/2_ and 4f_7/2_ doublets at binding energies positions of 74.53 eV and 71.35 eV, respectively^37^ (Fig. 3C). Importantly, the negative shift in the Pt 4f scan peaks is mainly ascribed to the modification in the electronic structure of Pt due to the incorporation of Pd into its lattice after the successful formation of the PtPd NDs alloy. This shift in the Pt 4f scan can be clearly observed with comparative XPS of Pt 4f scans for Pt NDs/RGO and PtPd NDs/RGO before and after the incorporation Pd into the Pt nanodendrites, shown in Fig. S2. The negative shift in the Pt 4f peak and slight distance expansion between the Pt4f_7/2_ and Pt 4f_5/2_ domains in the PtPd NDs/RGO catalyst can also be ascribed to the fact that Pd is slightly more electronegative compared to Pt and tends to transfer electrons from Pd to Pt upon alloying. Due to this electron transfer trend (from Pd to Pt), the electron densities on Pt increases. Therefore, the high electron density on Pt also lowers the binding energy value of Pt electrons. The whole phenomenon of electronic modifications results in a negative shift of the Pt 4f peak, influencing the spin–orbit splitting and causing distance expansion in between the Pt 4f_7/2_ and Pt 4f_5/2_ domains. The Pd 3d XPS scan showed a split into two spin–orbit doublets, 3d_5/2_ and 3d_3/2_, at binding energy positions of 334.95 eV and 340.21 eV, respectively, suggesting the metallic Pd(0) state in the NDs alloys^38^ (Fig. 3D).

The electrocatalytic MOR performance for the synthesized catalyst was tested by half-cell testing (three-electrode setup) *via* cyclic voltammetry (CV) and linear sweep voltammetry (LSV) in 0.1 M HClO_4_ + 1 M CH_3_OH solution. The screening of a suitable low-content loading amount for Pd incorporation into Pt ND was examined with four different Pd nominal loadings (such as, 0.5 wt%, 1 wt%, 2 wt% and 3 wt%) to maintain the cumulative 5 wt% Pt–Pd in the PtPd NDs/RGO catalyst (actual wt% were also measured and confirmed by ICP-MS analysis) and tested for MOR. The results revealed that loading 1 wt% of Pd into 4 wt% of Pt exhibited enhanced MOR and outperformed the other compositions. The linear sweep voltammetric (LSV) curves (If_max_, *i.e.*, forward scan) up to 0.70 V *vs.* RHE for all Pt-based ND with different incorporations of Pd loading are presented in Fig. S3 (in the SI file). The lower or negligible increase in the MOR performance of the catalyst with less than 1 wt% incorporation is ascribed to there being insufficient Pd sites for enhancing the electrocatalytic performance of the PtPd NDs/RGO catalyst. In cases with more than 1 wt% incorporation of Pd to the catalysts, the MOR reaction performance gradually decreased. This decrease in the MOR performance can be attributed to the common factor of an excessive Pd proportion in the PtPd NDs/RGO catalysts suppressing the Pt active sites that were more favourable for MOR.^39,40^ The content-performance relation for enhanced methanol oxidation activity at an optimized proportion of Pd with Pt in the PtPd NDs/RGO catalyst can also be ascribed to the favourable presence of both metals. The higher Pt-proportion in the catalyst clearly facilitated the fast cleavage of crucial C–H bonds, followed by smooth oxidation steps of methanol into CO_2_. The incorporated low-content Pd into Pt also optimized the adsorption energies of the intermediates by Pt d-band tuning. Moreover, the bi-functional mechanism enhanced the MOR, assisting the unblocking of Pt-active sites in PtPd NDs. In contrast, the higher loading of Pd adversely affected MOR by suppressing the Pt-sites and making the C–H bond cleavage more inefficient. The higher proportion of dominating Pd sites in the catalyst also accumulates un-oxidized CO_ads_ on the surface, which decreases the performance of the catalyst. Based on the pre-screening study, further deep analysis and testing were performed with PtPd NDs/RGO with optimized (1 wt%) low-content Pd incorporation.

The optimized composition catalyst referred as PtPd NDs/RGO in the whole study was considered for detailed MOR measurements. The electrochemical active surface area (ECSA) of the catalysts was calculated with the typical cyclic voltametric (CV's) response of the catalysts, shown in Fig. S4. The calculated ECSA for the Pt NDs/RGO and PtPd NDs/RGO catalysts was 374.08 cm^2^ mg_Pt_^−1^ and 491.58 cm^2^ mg_Pt_^−1^, respectively. These ECSA values were calculated by integrating the area bound by the H_upd_ curve and the baseline in the range of 0.05–0.4 V (from CV's). The ECSA value for the Pt-based alloy catalysts can be determined by QH = *mq*, where QH represents the total electric charge of H_upd_ on Pt-based materials, *m* is the catalyst loading on the GCE electrode in grams (g), and the charge of each actual active area is assumed to be 210 mC cm^−2^.^39^ The apparent increase in ECSA for Pt NDs/RGO after incorporation of Pd in PtPd NDs/RGO also suggests the enhanced surface area of the catalysts with more available active sites responsible for MOR electrocatalysis.

**Fig. 4:**
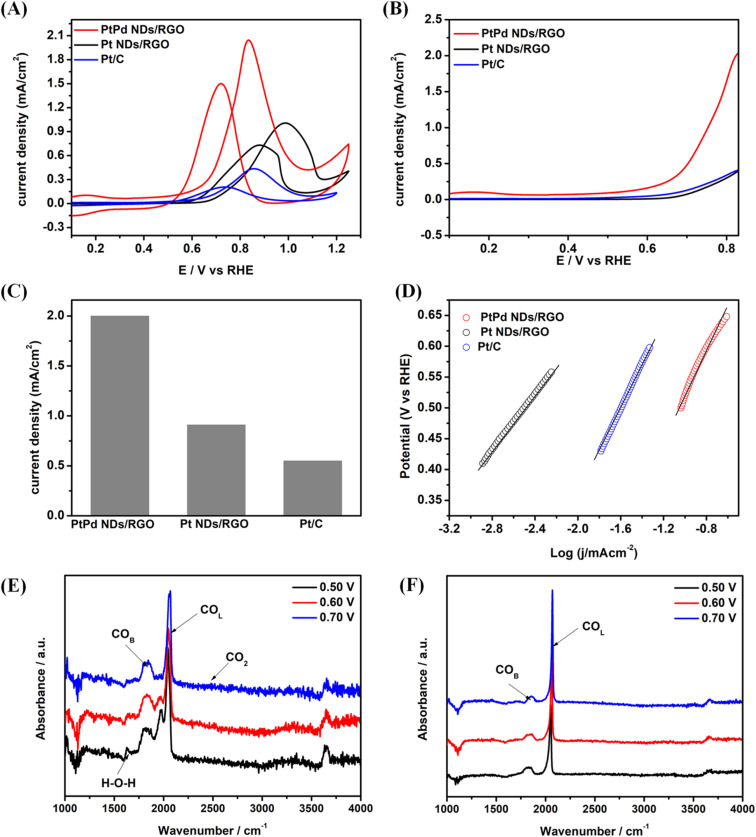
MOR analysis for (A) the catalysts PtPd NDs/RGO, Pt NDs/RGO and Pt/C; (B) the LSV result for the comparison of the onset potential for the catalysts PtPd NDs/RGO and Pt NDs/RGO, (C) comparison of the specific activities of the catalysts taken at the If_max_ from MOR curves and (D) the corresponding Tafel plots for MOR activities. The MOR analysis was carried out in 0.1 M HClO_4_ + 1 M CH_3_OH solution with N_2_ purging at a scan rate of 50 mV s^−1^. Electrochemical *in situ* FTIR (time-resolved) spectra recorded during the methanol oxidation at different potentials in 0.1 M HClO_4_ + 1 M CH_3_OH on the (E) PtPd NDs/RGO catalyst and (F) Pt NDs/RGO catalyst.

The electrochemical CV results of the methanol oxidation reaction for the PtPd NDs/RGO catalyst exhibit the typical trends with two peaks (Fig. 4A). The forward peak is designated for the adsorption and chemisorption of methanol molecules. The backward peak is ascribed to the further chemisorption of the adsorbed intermediate species on the catalyst surface to unblock the metal active sites. The proposed straightforward mechanism for MOR on PtPd NDs/RGO can be referred to as the methanol molecules actively adsorbed on the active metal sites on the catalyst. These adsorbed methanol molecules are converted into the final product following the formation of intermediate species leaving the active sites. Among these intermediate species, CO_ads_ are supposed to strongly adsorb on Pt-sites and further block the methanol oxidation process. These adsorbed CO_ads_ species were further oxidized into CO_2_ in the presence of adjacent OH_ads_ species on more oxophilic Pd-sites in the catalyst, which came from the dehydrogenation of water molecules. In addition, due to the formation of PtPd alloys, the structural modifications in Pt d-bands helped to weaken the Pt-CO_ads_ strong interactions to unblock these poisonous species.

To support the above-explained bifunctional mechanism in the PtPd NDs/RGO catalyst, the MOR performance of the ND catalyst was compared to that of the Pt NDs/RGO catalyst in the absence of Pd. The enhanced methanol oxidation can be clearly observed in higher current density with specific activities of 2 mA cm^−2^ and 0.91 mA cm^−2^ and mass activities of 998.5 mA mg_Pt_^−1^ and 356.7 mA mg_Pt_^−1^ for PtPd NDs/RGO and Pt NDs/RGO, respectively. The onset potential for the methanol oxidation reaction was also shifted towards lower potential values for PtPd NDs/RGO at 0.5 V *vs.* RHE compared to that of Pt NDs/RGO at 0.58 V *vs.* RHE. These enhanced features for the higher current density and lower onset potential in the PtPd NDs/RGO catalyst suggest the favourable presence of Pd with Pt in the catalyst and introduced bifunctional mechanism in MOR. As the MOR possesses a six-electron transfer reaction for its completion, the presence of RGO in the catalyst played a significant role in the transfer of electrons from the support to the metal active sites. This phenomenon was further confirmed by comparing the MOR performance of the catalyst with that of the Pt/C (20 wt%) commercial catalyst. It can be clearly observed from the results that ND-based catalysts in the presence or absence of Pd alongside Pt outperform the Pt/C catalyst (Fig. 4A). A critical comparison of the onset potential and peak current density values (If_max_) for PtPd NDs/RGO, Pt NDs/RGO and Pt/C is also shown in Fig. 4B and C, respectively.

Furthermore, the kinetics of the methanol oxidation reaction was determined by means of Tafel plot from the MOR current density peaks of the catalysts. The plot generally gives the Tafel slope and exchanged current density (intercept) values ascribing the reaction mechanism and rate determining steps, respectively.^41,42^ A smaller value of the Tafel slope suggests the faster reaction rate at a given overpotential. The drawn Tafel plot for the catalysts, shown in Fig. 4D, gave the calculated Tafel slope values of Pt/C (377 mV dec^−1^), Pt NDs/RGO (292 mV dec^−1^) and PtPd NDs/RGO (218 mV dec^−1^). Along with these, the exchanged current density values for PtPd NDs/RGO were observed to be higher than that of Pt NDs/RGO and comparable to Pt/C. These observed trends in the kinetic evaluation of the MOR results with a lower Tafel slope and higher exchanged current density at equilibrium for PtPd NDs/RGO compared to that for Pt NDs/RGO suggested the significantly enhanced MOR performance of the nanodendrites catalyst after Pd incorporation. Moreover, the electrochemical impedance spectroscopy (EIS) tests were conducted for evaluation of the electron transfer trends in the catalysts during MOR, as shown in Fig. S5. The typical EIS results exhibit the real impedance (*Z*′) along the *x*-axis for ohmic resistance and imaginary impedance (−*Z*′′) for the capacitive behaviour of the catalysts. The observed EIS curves for the catalysts allowed for the conclusion that the smaller arc area of the peaks represented a lower charge transfer resistance for PtPd NDs/RGO compared to those of Pt NDs/RGO and Pt/C during MOR; hence, with possible low or negligible CO poisoning on the catalyst, as shown in Fig. S5(A). Moreover, the EIS Nyquist fitting has been proposed and determined for the obtained EIS results, given in Fig. S5(B). The designated areas are ascribed as (*R*_b_) for bulk resistance, (*R*_SEI_) and (CPE_SEI_) for charge transfer resistance and double layer capacitance, respectively, and (*R*_ct_) and (CPE_electrode_) for the resistance and capacitance of the interfacial layers, respectively. The corresponding fitted circuit is given in Fig. S5(A). Based on the fitted EIS Nyquist plot and circuit, it can be clearly observed that the PtPd NDs/RGO catalyst experienced the lowest charge transfer resistance compared to the Pt NDs/RGO and Pt/C catalysts. Thus, the EIS results support the claim of an enhanced electron transfer phenomenon in the PtPd NDs/RGO catalyst with higher electrocatalytic MOR activity.

The claim of low-CO poisoning on PtPd NDs/RGO compared to the Pt NDs/RGO catalyst was also confirmed by CO-stripping experiments. Shown in Fig. S6, the first scan during the CO-stripping experiments revealed the absence of the H_upd_ region in both catalysts due to the strong adsorption of CO on its active sites. As the potential reached the range for CO-oxidation for the catalysts, sharp CO-stripping peaks appeared for PtPd NDs/RGO at lower potential compared to Pt NDs/RGO. After successful oxidation of CO, the H_upd_ region normally appeared with the CVs of the catalysts in further scan, indicating complete removal of CO from active sites.^43^ This quick oxidation of CO at PtPd NDs/RGO suggested the un-blocking of the active sites assisted by the bi-functional mechanism due to the presence of Pd with Pt. Therefore, the key active sites responsible for the enhanced electrochemical behaviour of the PtPd NDs/RGO (optimized) catalyst can be ascribed to the following factors: platinum-rich sites acted as primary sites for methanol molecules adsorption and dehydrogenation, Pt–Pd interfaces facilitated CO tolerance and the bifunctional mechanism by generating electronic and geometric effects and the high indexed facets offered from the nanodendrite geometry enhanced the activation of methanol and oxidation of intermediates (*i.e.*, CO_ads_).

Spectroelectrochemical characterizations by *in situ* FTIR and online differential electrochemical mass spectrometry (DEMS) were conducted for the PtPd NDs/RGO and Pt NDs/RGO catalysts to reveal the mechanism of MOR and its conversion into the final product, respectively. The enhanced performance of the PtPd NDs/RGO with incorporated Pd into Pt NDs/RGO can be revealed from comparative analysis of the obtained *in situ* FTIR spectra for both the catalyst materials (Fig. 4E and F). In the IR spectra, the strong signals for the CO_ads_ species can be observed in the case of Pt NDs/RGO compared to PtPd NDs/RGO. The stretching vibration band for CO_B_ (bridge-bounded) is at a higher intensity in PtPd NDs/RGO compared to that of the Pt NDs/RGO catalyst. These observations in the obtained CO_ads_ bands confirm the weakly adsorbed CO poisonous species on the PtPd NDs/RGO catalyst can be actively removed and vacated from the active sites for further chemisorption process with enhanced performance of the catalyst. Moreover, the slight band signals around at 2343 cm^−1^ ascribed to CO_2_ more clearly appeared in the case of PtPd NDs/RGO. Along with these, the additional appearance of a broad band centered between 1600 and 1650 cm^−1^ is designated to the bending vibration of H–O–H in the PtPd NDs/RGO catalyst, suggesting the adsorption of water molecules and their dissociation on adjacent Pd-sites in the catalyst.^44–46^ Along with these adsorbed water molecules, an additional IR band signal at around 3300 cm^−1^ appeared for the PtPd NDs/RGO catalyst ascribed to the stretching vibration of O–H_ads_ from interfacial water dissociation associated with Pd-sites. This dissociation of water generates OH_ads_ adjacent to the CO_ads_ species, which react with each other and form CO_2_ to complete the methanol oxidation and thereby release the active sites for further electrocatalytic reaction.^44–47^

**Fig. 5:**
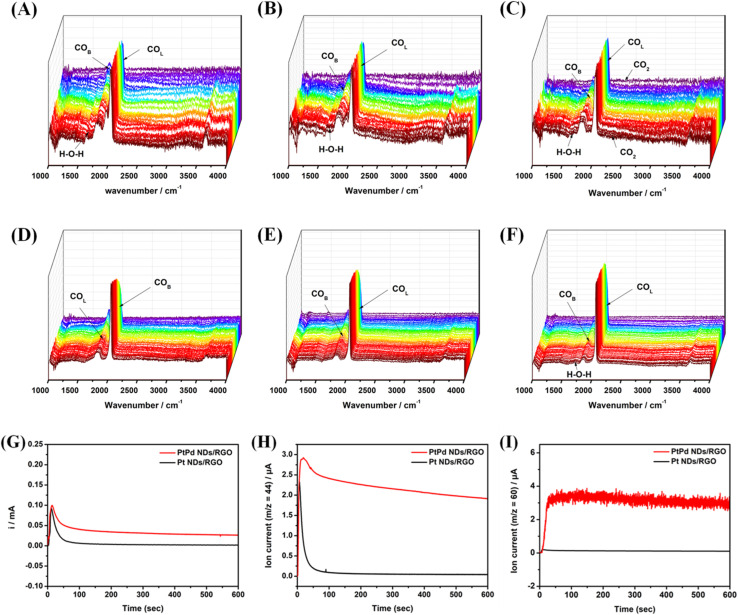
Electrochemical *in situ* FTIR (time-resolved) spectra recorded during the methanol oxidation for PtPd NDs/RGO (A) at 0.50 V, (B) at 0.60 V and (C) at 0.70 V and for Pt NDs/RGO (D) at 0.50 V, (E) at 0.60 V and (F) at 0.70 V *vs.* RHE in 0.1 M HClO_4_ + 1 M CH_3_OH, holding up to 50 seconds with one segment completing in 10 seconds. Online DEMS response at a potential of 0.70 V, time course of 600 s with (G) current *vs.* time plot, (H) reaction current and mass signal *m*/*z* = 44 from CO_2_ and (I) *m*/*z* = 60 for methyl formate for the PtPd NDs/RGO (red color) catalyst and Pt NDs/RGO (black color) catalyst.

For the time-resolved *in situ* FTIR spectra for PtPd NDs/RGO and Pt NDs/RGO, both catalysts were studied at potential values of 0.50 V, 0.60 V and 0.70 V *vs.* RHE holding up to 50 seconds with one segment completing in 10 seconds (Fig. 5A–F). In both catalysts, the band proportion appearing between the regions of 2050–2150 cm^−1^ and 1750–1950 cm^−1^ came from the CO_ads_ species being adsorbed on the active sites of the catalyst in the form of linearly-bounded (CO_L_) and bridge-bounded (CO_B_), respectively, during MOR. The CO_ads_ species that appeared on the active sites are further converted into CO_2_ after the complete chemisorption process. The FTIR bands around 1150–1450 cm^−1^ may be ascribed to the stretching vibrations from the intermediate species CHO and COOH.^44,45^ These *in situ* FTIR band trends suggested the direct methanol oxidation reaction mechanism on the catalyst, starting from the adsorption of methanol molecules on the active sites. These adsorbed methanol molecules are converted into the final product following the active intermediate formation pathway.

It has been found that from lower to higher potentials, changes in the IR peaks are clearly observed. At the lower potential value, the peak proportion for water dissociations (H–O–H) appeared for the PtPd NDs/RGO catalyst, suggesting the fast chemisorption and removal of the intermediate species at lower potentials. This may lead to the complete oxidation of methanol with the CO_2_ peak proportion at higher potential values. In comparison, for the Pt NDs/RGO catalyst, the H–O–H peaks tend to appear at higher potential values, suggesting the blocking or poisoning of the active sites at lower potential. This phenomenon can be correlated with the enhanced methanol oxidation of the catalyst, showing a quick oxidation of the intermediate species and unblocking the active sites for further chemisorption of the methanol molecules.

An online DEMS study was done to confirm the formation of the final product CO_2_, along with methaformate, after the complete and successful electro-oxidation of methanol (Fig. 5 G–I). This spectroelectrochemical investigation effectively measures the electro-oxidation product by the mass spectrometric (*m*/*z* = 44) ion current signals recorded and further converted into partial faradaic current for CO_2_ molecules.^48^ The recorded faradaic current associated with mass signals of CO_2_ (*m*/*z* = 44) as a function of reaction time revealed the complete oxidation of methanol molecules following the six-electron transfer reaction *via* active intermediate pathway.^49^ The chronoamperometric current (Fig. 5G) and the corresponding partial faradaic current for CO_2_ production (Fig. 5H) recorded at a potential of 0.70 V *vs.* RHE for PtPd NDs/RGO and Pd NDs/RGO suggest the enhanced electrocatalytic performance of the PtPd NDs/RGO catalysts. During methanol oxidation, the released/transferred electrons per CO_2_ molecule are ascribed to the six-electron (*n* = 6) transfer per methanol molecule conversion. As the CO_2_ ion current values recorded for PtPd NDs/RGO were six times greater than that of Pt NDs/RGO at the set potential, we assume the quick, smooth and enhanced conversion of methanol into the final product on the Pt-based NDs in presence of Pd.^48–50^ Moreover, the current signals for methaformate (*m*/*z* = 60), shown in Fig. 5I, suggest the simultaneous production of the methaformate species by oxidation of formic acid following the simple two-electron transfer reaction. The observed methaformate current signals clearly indicates that the methanol oxidation reaction follows the active-intermediate pathway, negating the effect of active site poisoning issues.^47,51^

**Fig. 6:**
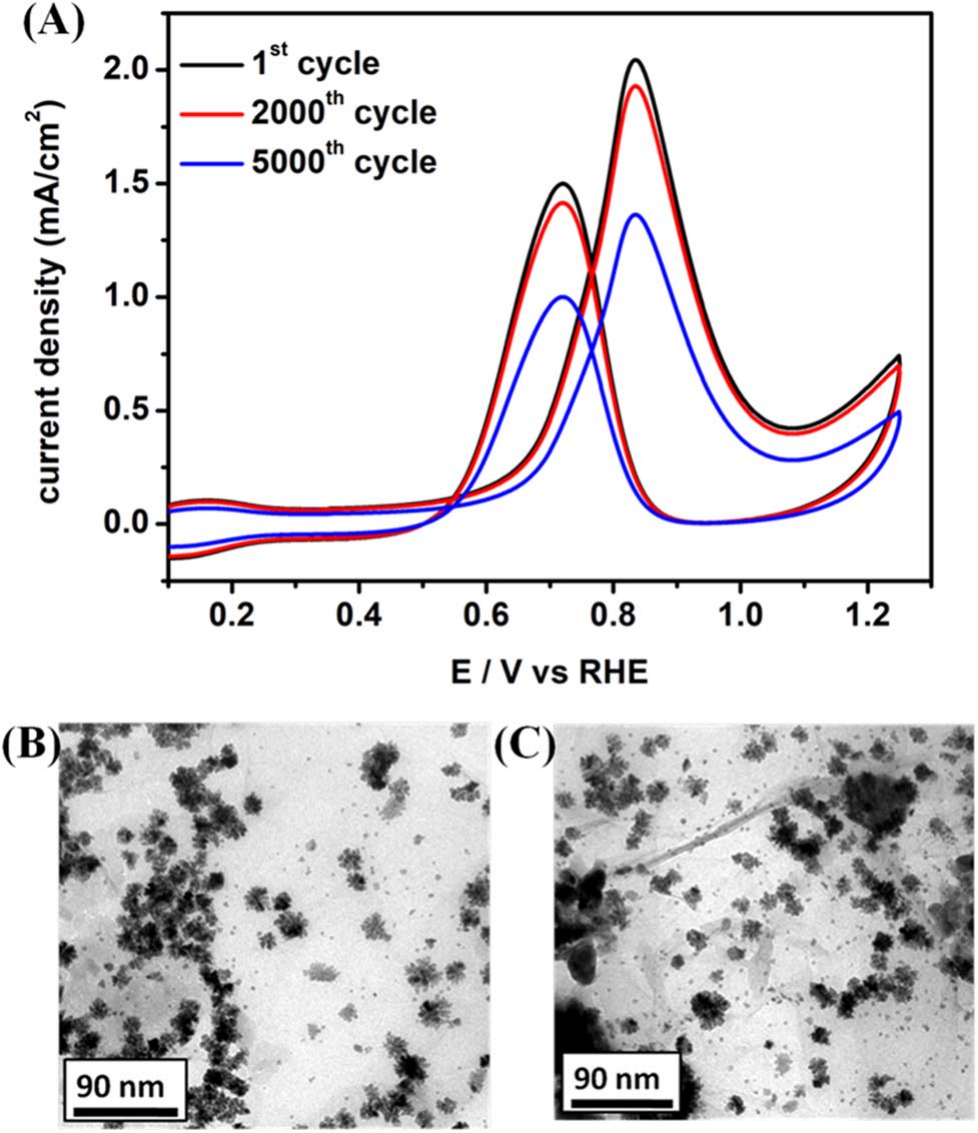
(A) Accelerated durability test (ADT) for the PtPd NDs/RGO catalyst in the same environment at a scan rate of 100 mV s^−1^ for up to 2000 cycles and 5000 cycles, and morphological analysis for the PtPd NDs/RGO catalyst. (B) TEM image before ADT and (C) TEM image after ADT.

An accelerated durability test (ADTs) of the PtPd NDs/RGO catalyst was also conducted to evaluate the stability and durability of the catalyst material after working in harsh working conditions. The ADTs was performed with CV measurements in the same environment and medium (0.1 M HClO_4_ + 1 M CH_3_OH) at a scan rate of 100 mV s^−1^. After 5000 rounds of potential cycling for the methanol oxidation reaction, the catalyst showed significant retention in its actual performance. The recorded current densities for the catalyst show a retention of 95% after 2000 cycles and retention of 70% after 5000 cycles in its initial performance (Fig. 6A). In a more detailed analysis, the obtained current density values before and after ADTs at If_max_ have also been provided in the inset of Fig. 6A. The ADTs results revealed the durable nature of the catalyst by successfully dealing with the stability issues of the electrocatalysts, such as surface corrosion, agglomeration of particles and loss of metal active sites.^52^ Moreover, these claimed properties of the durable catalyst material were confirmed by re-evaluating the morphological analysis. The TEM analysis of the catalyst after ADTs was performed and compared to that before the ADT measurements. Shown in Fig. 4B and C, the ND retained their original shapes and stable dispersion even after the ADTs. In addition, no agglomeration of the ND was observed after the ADTs, clearly showing the morphological stability and performance durability.

**Fig. 7:**
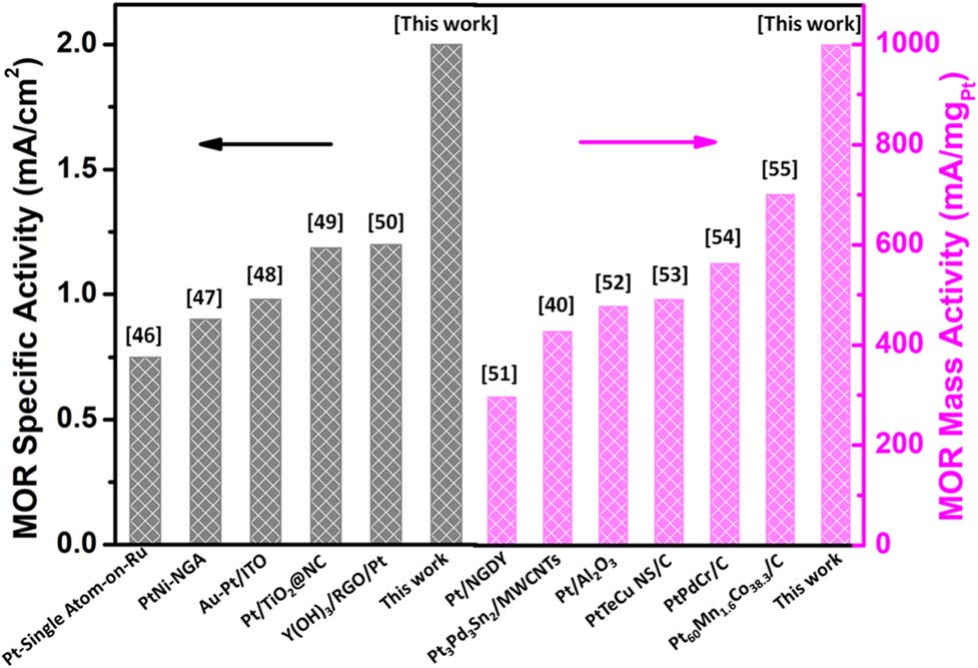
Comparison of the methanol oxidation reaction (MOR) specific activity of the PtPd NDs/RGO (this work) catalyst with those of similar types of Pt-based bimetallic catalysts reported in the literature.

The enhanced methanol oxidation reaction performance of the catalyst developed in this work has also been compared to that of different Pt-based bimetallic alloys with different shapes and on different support materials reported in the literature, presented in Fig. 7^47,53–62^ and Table S2 (in the SI file). The enhanced performance of the catalyst was mainly ascribed to the collective features of the nanodendrite shape and low-loading of precious metals.^63–65^ This critical comparison among the reported catalysts clearly shows the remarkable performance of the PtPd NDs/RGO catalyst in contrast to that of similar kinds of catalysts in the methanol oxidation reaction.

## Conclusions

We have been successful in developing a high performance, durable and low-cost electrocatalyst for the methanol oxidation reaction. The study was designed to form low-content alloyed PdPt ND by co-precipitation single-step method. PtPd ND was uniformly dispersed and loaded on RGO. The screening showed optimal Pd loading with 1 wt% of Pd and 4 wt% of Pt as the catalyst PtPd NDs/RGO. The TEM analysis confirmed the ND morphology and ND's uniform dispersion on RGO. The HRTEM analysis, fast Fourier transform (FFT), inverse FFT images and line spectra confirmed the lattice plane information of the Pt–Pd dendrites in alloy form and supported the XRD results. All of the physical characterizations together with the XPS analysis confirmed the successful incorporation of Pd into Pt as the PtPd homogenous alloy in the PtPd NDs/RGO catalyst.

The electrochemical characterizations for the methanol oxidation reaction were carried out initially using a half-cell testing method and later using spectroelectrochemical techniques (*i.e.*, *in situ* FTIR and online DEMS). The half-cell testing revealed the enhanced MOR activity for PtPd NDs/RGO compared to that of Pt NDs/RGO and commercial Pt/C (20 wt%). As the catalyst contained 4 times lower wt% of Pt-group metals compared to the commercial Pt/C, its specific activity for methanol oxidation was observed to be 4 times greater than that for the Pt/C catalyst. The *in situ* FTIR analysis revealed the conversion of methanol molecules into a final product, followed by an active intermediate pathway *via* quick removal of poisonous intermediates such as CO_ads_ from active metal sites. The catalyst smoothly obeyed the six-electron transfer reaction *via* the adsorption of methanol to CO_2_, followed by the appearance of CHO, COOH and CO at Pt-sites. The appearance of interfacial water species, *i.e.*, OH_ads_ at adjacent Pd-sites, facilitated the oxidation of CO_ads_ and unblocking of the Pt-active sites for further adsorption and chemisorption of methanol. No additional IR bands were observed associated with methanolic fragments from the poisonous intermediate pathway, suggesting the straightforward, smooth conversion of methanol into CO_2_. The online DEMS analysis further confirmed the production of the final product with detected partial faradaic CO_2_ ion current. This differential electrochemical mass spectrometry also revealed the higher CO_2_ ion current for PtPd NDs/RGO. The outcomes from this study gave a series of benefits in the venue of electrocatalysis and energy conversion, such as introducing high-performance catalysts outperforming the high-cost commercial Pt/C and a facile strategy to develop low-cost Pt-group metal-based catalysts for fuel cells.

## Conflicts of interest

There are no conflicts to declare.

## Supplementary Material

NA-007-D5NA00672D-s001

## Data Availability

Data sharing is not applicable to this article as no datasets were generated or analysed during the current study. The additional results supporting this article have been included as part of the supplementary information (SI). Supplementary information is available. See DOI: https://doi.org/10.1039/d5na00672d.
