# Supplementary material for: Lattice add-ons with low-content Pd incorporated into Pt nanodendrites revealed active methanol oxidation via the fast removal of poisonous intermediates
Source: Nanoscale Adv. 2025 Oct 13;7(21):6796–807. doi: 10.1039/d5na00672d (PMC12516348; doi:10.1039/d5na00672d)
Supplement: NA-007-D5NA00672D-s001 [file NA-007-D5NA00672D-s001.pdf]

Supplementary Information (SI) for Nanoscale Advances.
This journal is © The Royal Society of Chemistry 2025

Electronic Supporting Information (ESI) File

Lattice Add-Ons with Low-Content Pd into Pt Nanodendrites Revealed Active Methanol Oxidation via Fast Removal of Poisonous Intermediates

Ammar Bin Yousaf[1,*], Asad Ali[2] and Peter Kasak[1,*]

[1]Center for Advanced Materials, Qatar University, Doha 2713, Qatar

[2]School of Materials Science and Engineering, Hefei University of Technology, Hefei, 230009, P.R. China

[*] Corresponding Authors E-mails: ammar@mail.ustc.edu.cn / ammar.chemist18@gmail.com (A.B. Yousaf) and peter.kasak@qu.edu.qa (P. Kasak)

Experimental:

Chemical and Reagents

Graphite, H_2PdCl_4 (99.9% metal basis) was from Alfa Aesar, K_2PtCl_6 (40%) was purchased from Fluka, hexadecyl trimethyl ammonium bromides (CTAB), L-ascorbic acid, $HClO_4$. Other reagents were purchased from Sinopharm Chemical Reagent Co., Ltd. All reagents were analytical reagent grade. Functionalized graphene oxide (GO) was synthesized according to modified previous literature by Hummer's method [1].

Synthesis of PtPd NDs/RGO catalyst

Pre-synthesised functionalized GO was added to Mili-Q water (1mg/1mL) and homogenously dispersed by ultrasonication. To this dispersion, the 10 mL of 1.25×10^{-2} M CTAB was added with continuous stirring at room temperature. Subsequently, the Pt (K_2PtCl_6) and Pd

(H_2PdCl_4) precursors with nominal loading of 4 wt% Pt and 1 wt% of Pd with cumulative noble metal loadings of 5 wt% in catalyst were added to this dispersion dropwise. The reaction mixture was stirred and heated upto 95 ^oC for 30 minutes. Then, freshly prepared 0.1 M L-ascorbic acid (80 mL) was added dropwise under constant stirring and heating. After addition, the reaction mixture was stirred for a further 15 minutes. As the PtPd NDs alloy particles developed on functionalized GO, L-ascorbic acid acted also as a reducing agent for transforming GO into RGO. The catalyst material (PtPd NDs/RGO) was centrifuged, washed with water, and dried in a vacuum oven overnight for further characterization and application. This catalyst was chosen for characterization due to its best performance in MOR, as described and discussed below. For comparison, Pt NDs/RGO catalyst without the incorporation (presence) of Pd was synthesized with a similar method by taking the nominal composition Pt (5 wt%) in the catalyst. In addition, the series of catalysts materials with three different wt% compositions of Pd (0.5 wt%, 2 wt% and 3 wt%) were also synthesized other than the best performance catalyst material with 1 wt% by following the same synthesis procedures. This brief library of catalysts helped to evaluate the optimized performance catalyst for electrocatalytic performance.

Characterizations

TEM images and high-resolution transmission electron microscopic (HRTEM) images were carried out on a JEM-2100F field emission electron microscope at an accelerating voltage of 200 kV. The high-angle annular dark-field scanning transmission electron microscopy (HAADF-STEM) images were taken on a JEOL JEM-ARF200F atomic resolution analytical microscope. The powder X-ray diffraction (XRD)patterns of the catalyst were tested on a Rigaku/Max-3A X-ray diffractometer with Cu Kα radiation (λ = 1.54178 A), the operation voltage and current were maintained at 40 kV and 200 mA, respectively. The X-ray

photoelectron spectroscopy (XPS) was performed at a Perkin-Elmer RBD upgraded PHI-5000C ESCA system.

Electrochemical measurements

The electrochemical measurements for half-cell testing were performed with a voltammetry technique and carried out with a CHI720Dand Gamry 600 electrochemical workstation. The glassy carbon electrode (GCE) was used as the working electrode. The Ag/AgCl (in 3 M KCl, aq.) combination, isolated in a double-junction chamber and a Pt coil were used as the reference and counter electrodes, respectively. All the measurements were performed in electrochemical experiments with respect to the standard values of reversible hydrogen electrode (RHE). Electrochemical experimental work was done using the potential cycling method.

Before each electrochemical experiment, GCE (0.196 cm^2 geometric surface area) was first polished on polishing cloth with alumina slurries (Al_2O_3, 0.05 mm) to obtain a mirror finish. The catalyst-coated GCE working electrode was prepared as 10µL of 2 mg/mL suspension in ethanol + 5 µL of Nafion (0.1 wt % in water) and was drop-coated on the polished electrode surface by a micropipette(with catalyst loading of 0.1 mg/cm^2). Whereas, the loading of the catalyst on GCE was calculated as followed,

Metal loading = $\left(0.1\frac{mg}{cm^2_{geo}}\right)$ (geometric surface area, 0.196) (metal %)

According to this calculation, 0.000980 mg/cm^2_{geo} and 0.000784 mg/cm^2_{geo} were determined for Pt NDs/RGO and PtPd NDs/RGO catalysts, respectively.

Electrochemical in situ FTIR reflection spectroscopy measurements were carried out on a Nexus 870 spectrometer (Nicolet) equipped with a liquid nitrogen-cooled MCT-Adetector. A

CaF_2 prism was used as the IR window. An IR cell was with a thin layer configuration between the electrode and the IR window was approached by pushing the electrode against the window before FTIR measurement. The time-resolved in-situ FTIR spectra were collected using multi-stepped FTIR spectroscopy (MSFTIR) procedures, and the spectra were taken at 0.50 V, 0.60V and 0.70 V reaction potentials (vs RHE). All spectra are obtained with a resolution of4 cm^{-1} and 1 spectrum/s. The spectra are presented in absorbance mode, $lg(R_0=R)$, where R_0 and $R$are the reflectance at reference and sample potential, respectively. Whereas the DEMS setup used in this study is a HidenHPR-40 DSA Bench top-membrane inlet gas analysis system, mass signals are collected at 20 points/s. The mass signal for CO_2 produced has been calibrated by oxidative stripping of a saturated CO adlayer pre-adsorbed at 0.06 V, mass calibration constant "$k = QF/Q$" mass is found to be 3.65×10^6 mA/Torr.

Table S1: ICP-MS results for Pt & Pd loading measurements of nanodenderites alloys after different loadings/incorporation of Pd into Pt NDs/RGO catalysts

**Catalyst**	**ICP-MS measurements**					
	**Pt (mol)**	**Pd (mol)**	**Pt (wt%)**	**Pd (wt%)**	**Pt (wt%)**	**Pd (wt%)**
			**Actual Loadings**		**Nominal Loadings**	
Pt NDs/RGO	5.352×10^{-6}	0	4.86	0	5	0
PtPd NDs/RGO (with 0.5 wt% Pd)	4.817×10^{-6}	0.297×10^{-6}	4.13	0.42	4.5	0.5
**PtPd NDs/RGO (with 1 wt% Pd)**	$\mathbf{4.291\times10^{-6}}$	$\mathbf{0.689\times10^{-6}}$	**3.93**	**0.94**	**4**	**1**
PtPd NDs/RGO (with 2 wt% Pd)	2.130×10^{-6}	1.280×10^{-6}	2.92	1.89	3	2
PtPd NDs/RGO (with 3 wt% Pd)	3.212×10^{-6}	1.960×10^{-6}	1.91	2.90	2	3

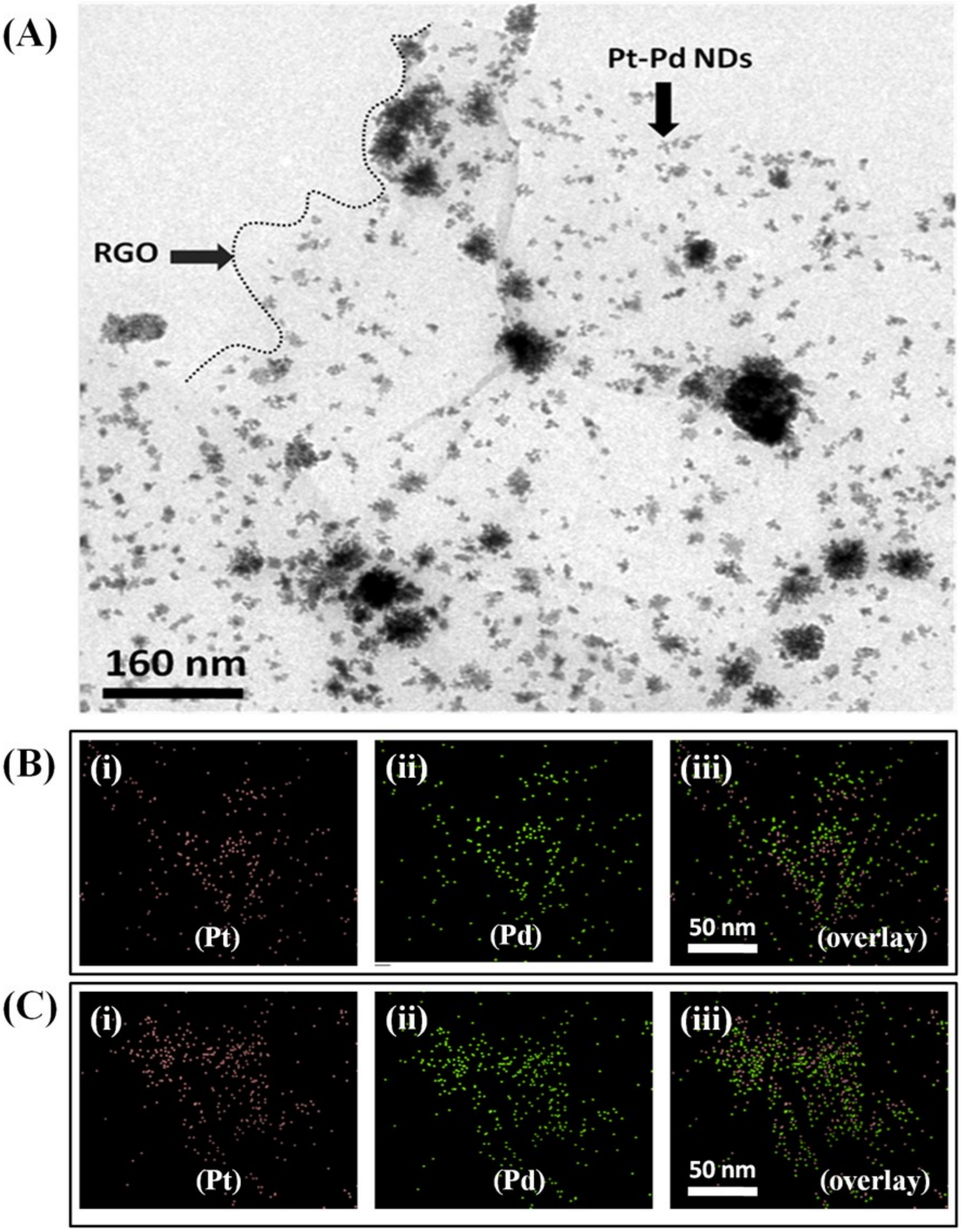

Figure S1: (A) TEM image for PtPd NDs/RGO catalyst; Comparative HAADF-STEM element mapping analysis for PtPd NDs/RGO **(B)** catalyst containing 2 wt% Pd and **(C)** catalyst containing 3 wt% Pd with specific area considered for the presence of **(i)** Pt **(ii)** Pd and **(iii)** the combined overlay image.

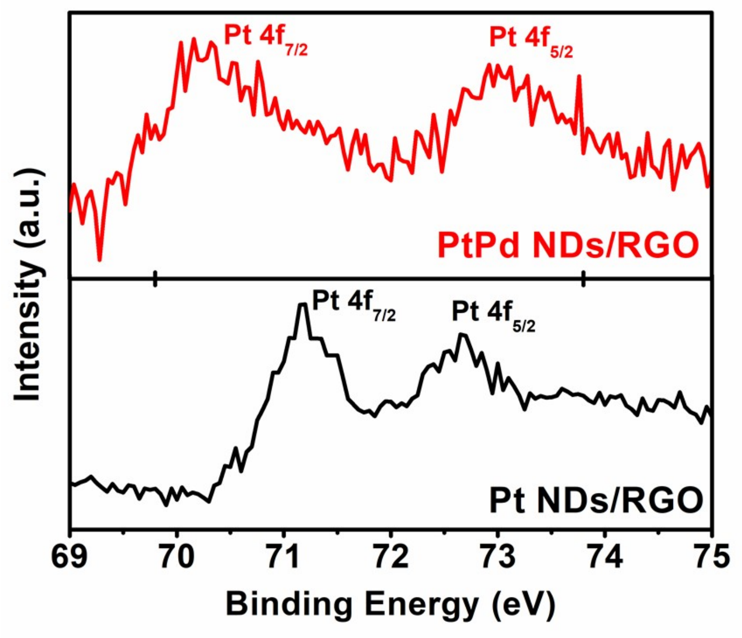

Figure S2: High resolution Pt 4f scans for Pt NDs/RGO and PtPd NDs/RGO catalysts

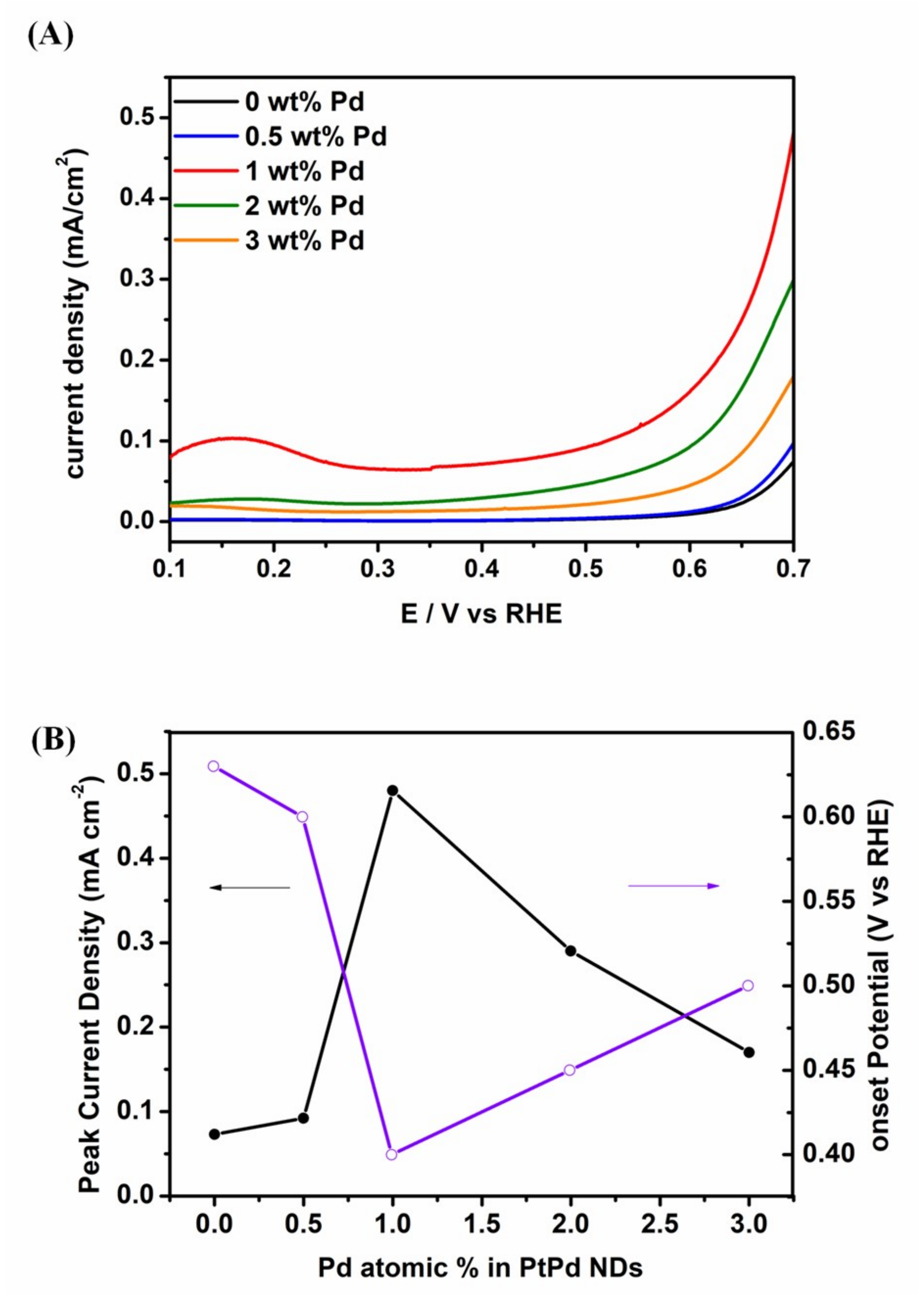

Figure S3: (A) Polarization curves for methanol oxidation reaction of catalyst optimizations with different loadings of Pd into Pt NDs/RGO, the presented forward scan (If) representing the comparison for onset potentials and peak current densities, the LSV scan is taken in 0.1 M $HClO_4$ + 1 M CH_3OH, (B) quantitative analysis for the comparison of peak current densities and onset potentials of the catalysts with different Pd loadings in PtPd NDs/RGO catalyst

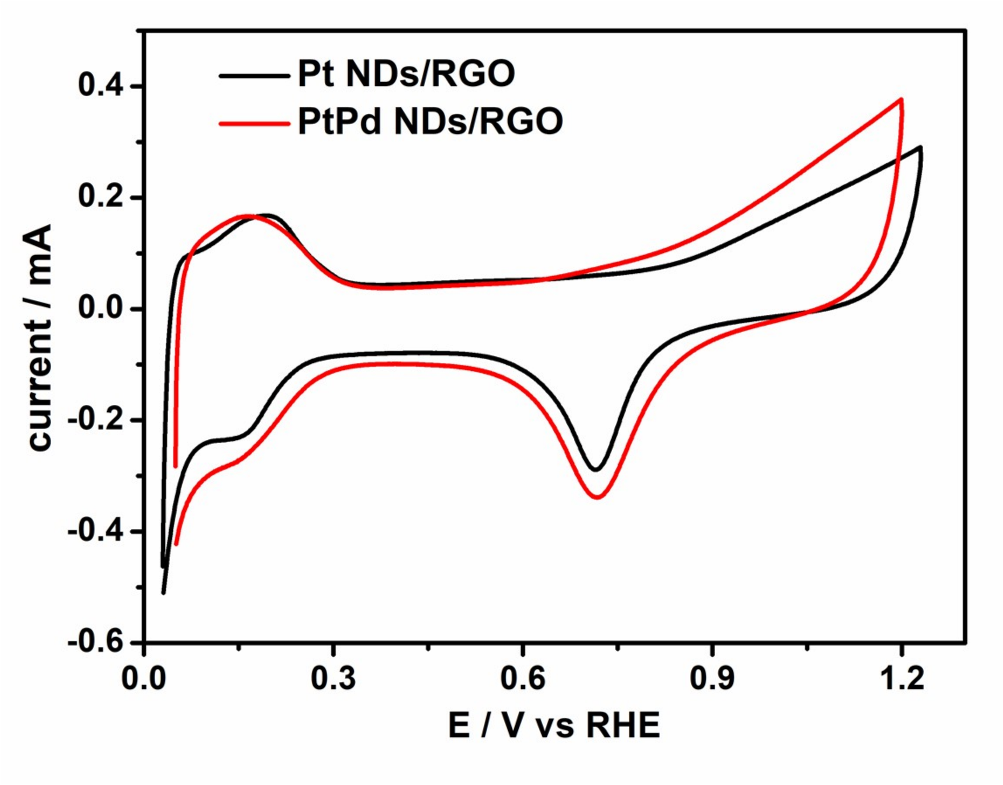

Figure S4: Cyclic voltammetric (CV) profiles for Pt NDs/RGO and PtPd NDs/RGO catalysts recorded in 0.1 M HClO4 at 50 mV/s scan rate

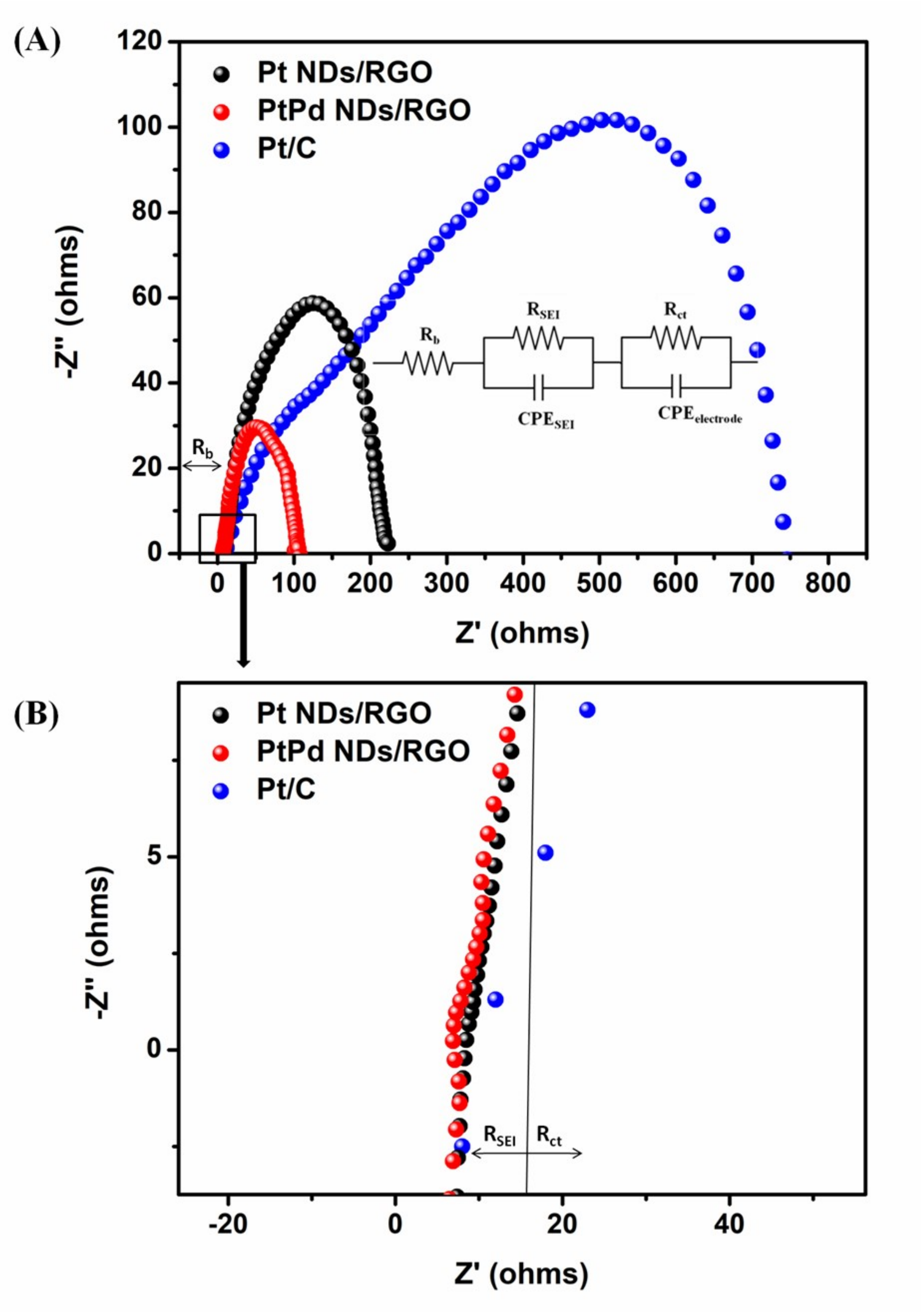

Figure S5: (A) EIS Nyquist plots of the studied catalysts compared to that of Pt/C, **(B)** zoom view of EIS plot for Nyquist fitting, recorded in 0.1 M $HClO_4$ + 1 MCH_3OH solution at 0.50 V vs. RHE

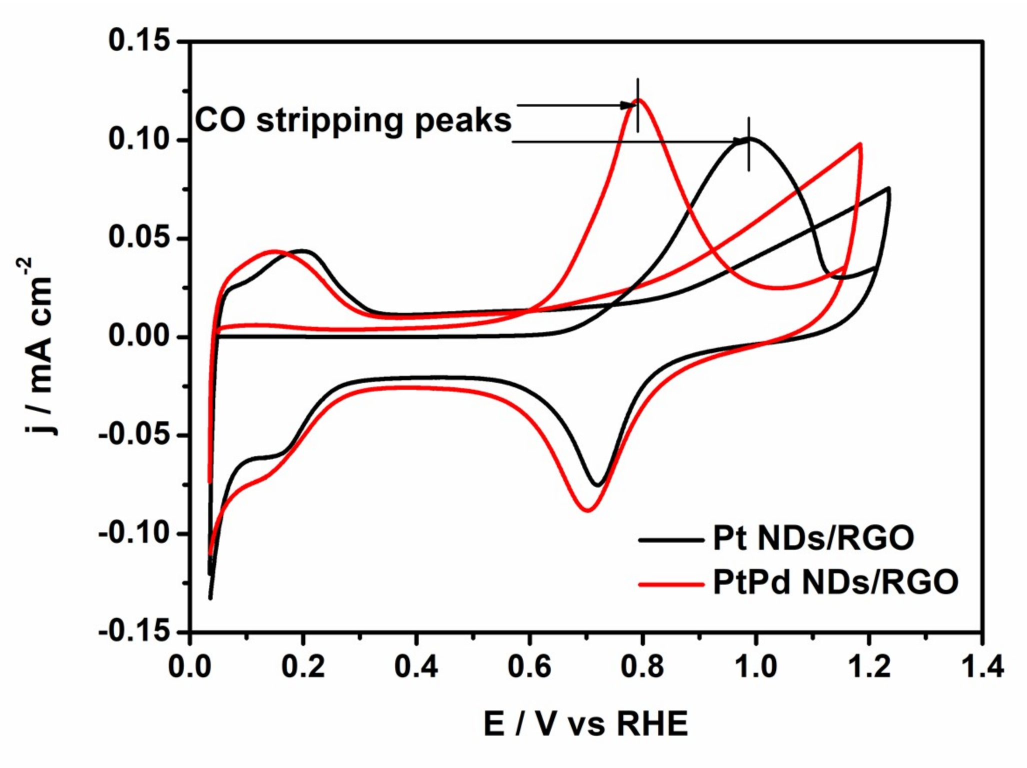

Figure S6: CO stripping curves for the PtPd NDs/RGO and Pt NDs/RGO (pre-adsorbed CO) catalysts in 0.1M $HClO_4$ solution at a scan rate of 50 mV/s vs RHE

Table S2: Comparison of methanol oxidation reaction (MOR) of PtPd NDs/RGO (this work) catalyst with those state-of-the-art methanol oxidation reaction electrocatalysts reported in the literature

Catalyst	MOR Activity	Reference
PtPd NDs/RGO	2 mA/cm^2 (specific activity)	This work
PtPd NDs/RGO	998.5 mA/mg_{Pt} (mass activity)	This work
Single-Pt-atom-on-Ru	0.75 mA/cm^2	[2]
PtNi-NGA	0.9 mA/cm^2	[3]
Au-Pt/ITO	0.98 mA/cm^2	[4]
Pt/TiO_2@NC	1.186 mA/cm^2	[5]
$Y(OH)_3$/RGO/Pt	1.2 mA/cm^2	[6]
Pt/NGDY	296 mA/mg_{Pt}	[7]
$Pt_3Pd_3Sn_2$/MWCNTs	426 mA/mg_{Pt}	[8]
Pt/Al_2O_3	476 mA/mg_{Pt}	[9]
PtTeCu NS/C	490 mA/mg_{Pt}	[10]
PtPdCr/C	562mA/mg_{Pt}	[11]
$Pt_{60}Mn_{1.6}Co_{38.3}$/C	700 mA/mg_{Pt}	[12]

References:

[1] W.S. Hummers, R.E. Offeman, *J. Am. Chem. Soc.*, 1958, **80**, 1339.

[2] A. R. Poerwoprajitno, L. Gloag, J. Watt, S. Cheong, X. Tan, H. Lei, H. A. Tahini, A. Henson, B. Subhash, N. M. Bedford, B. K. Miller, P. B. O'Mara, T. M. Benedetti, D. L. Huber, W. Zhang, S. C. Smith, J. J. Gooding, W. Schuhmann, R. D. Tilley, *Nat. Catal.* 2022, **5**, 231.

[3] Xu Chen, Jinyu Zhao, Jie Lian and Xiaomin Wang, *Green Chem.*, 2023, **25**, 3198-3207.

[4] H. Shen, M. Wang, W. Zhang, Y. Zhang, W. Wang, X. Cao, *J. Alloys and Comp.* 2022, **895**, 162581.

[5] J. Liu, L. Xu, X. Li, *Coll. Surf. A: Physicochem. Eng. Aspects*, 2022, **655**, 129986.

[6] K. Nubla , N. Sandhyarani, Mat. Res. Bull. 2023, **165**, 112304.

[7] L. Hui, Y. Xue, C. Xing, Y. Liu, Y. Du, Y. Fang, H. Yu, B. Huang, Y. Li, *Adv. Sci.* 2022, **9**, 2104991.

[8] S. Lyssenko, D. Kashyap, H. Teller, M.G. Gebru, A. Schechter, *Energy Fuels,* 2024, **38**, 19847−19859.

[9] E. Ponticorvo, M. Iuliano, C. Cirilloa, M. Sarno, *Chem. Eng. Trans.* 2023, **101**, 79-84.

[10] K. Dong, H. Dai, H. Pu, T. Zhang, Y. Wang, Y. Deng, *Appl. Surf. Sci.* 2023, **609**, 155301.

[11] K. Peng, N. Bhuvanendran, S. Ravichandran, W. Zhang, Q. Ma, L. Xing, Q. Xu, L. Khotseng, H. Su, *Int. J. Hydrogen Energy*, 2022, **45**, 22752-22760.

[12] P. Deshpande, B.L.V. Prasad, *ACS Appl. Mat. Int.* 2023, **15**, 26554-26562.
